# Effects of *ccpA* gene deficiency in *Lactobacillus delbrueckii* subsp. *bulgaricus* under aerobic conditions as assessed by proteomic analysis

**DOI:** 10.1186/s12934-020-1278-7

**Published:** 2020-01-13

**Authors:** Guofang Zhang, Libo Liu, Chun Li

**Affiliations:** Key Laboratory of Dairy Science, College of Food Science, Northeast Agricultural University, Harbin, 150030 China

**Keywords:** *Lactobacillus delbrueckii* subsp. *bulgaricus*, Catabolite control protein A, Stress response, Proteomics, Aerobic growth

## Abstract

**Background:**

Aerobic growth provides benefits in biomass yield and stress tolerance of *Lactobacillus delbrueckii* subsp. *bulgaricus* (*L. bulgaricus*). Catabolite control protein A (CcpA) is a master regulator involved in the aerobic and anaerobic growth, metabolic production and stress response in *L. bulgaricus*, but its potential molecular mechanisms remains unclear. The aim of this study is to elucidate the role of CcpA in *L. bulgaricus* in aerobic growth at the proteomic perspective.

**Results:**

The differential proteomic analysis was performed on the *L. bulgaricus* ATCC11842 and its *ccpA* inactivated mutant strain using iTRAQ technology. A total of 132 differentially expressed proteins were obtained, among which 58 were up-regulated and 74 were down-regulated. These proteins were mainly involved in the cellular stress response, carbohydrate and energy metabolism, amino acid transport and protein synthesis, genetic information processing. Moreover, inactivation of *ccpA* negatively affected the expression of key enzymes involved in glycolysis pathway, while it enhanced the expression of proteins related to the pyruvate pathway, supporting the conclusion that CcpA mediated the shift from homolactic fermentation to mixed acid fermentation in *L. bulgaricus.*

**Conclusions:**

Overall, these results showed that the role of CcpA in *L. bulgaricus* as a pleiotropic regulator in aerobic metabolism and stress response. This proteomic analysis also provide new insights into the CcpA-mediated regulatory network of *L. bulgaricus* and potential strategies to improve the production of starter and probiotic cultures based on the metabolic engineering of global regulators.

## Background

*Lactobacillus delbrueckii* subsp. *bulgaricus* (*L. bulgaricus*) belongs to the lactic acid bacteria (LAB), a heterogeneous group of microorganisms used as starter and/or adjuncts in the production of several fermented foods, including yoghurt and cheeses [1]. The use of *L. bulgaricus* in the dairy industry is, however, not without problems. The strain is exposed to different environments, including the human ingestion, and during preservation of starter and probiotic cultures, where it suffered several stresses (acid, heat, cold, oxidation, etc.) [2, 3]. To cope with these stresses and survive, *L. bulgaricus* has developed complex molecular response mechanisms, affecting many cellular processes such as carbohydrate and energy metabolism, cell membrane synthesis, transport, and bioadhesion [4–6].

The stress response in LAB is normally regulated by the induction of certain proteins such as heat shock proteins, cold shock proteins etc., and it is mainly mediated by the HrcA and CtsR repressors [7]. However, some common response mechanisms are triggered by different environmental stresses, thus suggesting a central role for global regulators. Among these, catabolite control protein A (CcpA) is one of the most important regulator since it is involved in the regulation of carbon catabolite repression (CCR) and various metabolic pathways in Gram-positive bacteria [8].

CcpA, which belongs to the LacI/Ga1R transcriptional regulatory family, contains 333 amino acids with a molecular weight of 37 kDa [8]. The role of CcpA in the control of metabolism and stress has been studied in several LAB, such as *Lactobacillus plantarum* [9–11], *Lactobacillus casei* [12, 13], and *Lactococcus lactis* [14, 15].

Some studies have demonstrated that the growth conditions under which aerobic (oxygen) and respiratory (aerobic growth in the presence of exogenous heme and/or menaquinone) growth improve the stress tolerance and biomass yield in several industrially important LAB [11, 16–20]. In *L. lactis*, inactivation of *ccpA* shifted homolactic fermentation to mixed acid fermentation under aeration conditions [14]. CcpA also plays a key role in respiration since it activated the repressor of the heme uptake preventing oxidative damage at the start of exponential growth of *L. lactis* [15]. In addition, CcpA positively regulated the expression of *hrcA* and *groESL* operons in *L. plantarum* [16], and aerobic growth improves stress tolerance. These results provide evidence for the role of CcpA in the regulation of aerobic metabolism, respiration and stress response. However, less is known about its role in *L. bulgaricus*. In addition, the scientific literature contains few studies concerning the role of the pleiotropic regulator CcpA at proteome level [21].

In previous work, we constructed a homologous *ccpA* deletion mutant strain of *L. bulgaricus* and performed a set of physiological and metabolic studies on the parental strain and the mutant strain [22]. Results showed that inactivation of *ccpA* significantly affected growth, metabolite production and stress tolerance.

To fully understand the central role of CcpA in modulating metabolism and stress *L. bulgaricus*, a thorough proteomic investigation on *L. bulgaricus* and its *ccpA* deletion mutant strain was reported in the present work. This integrative approach provides new insights into the cellular processes regulated by CcpA in *L. bulgaricus*.

## Results

### Initial proteomic data analysis

**Fig. 1:**
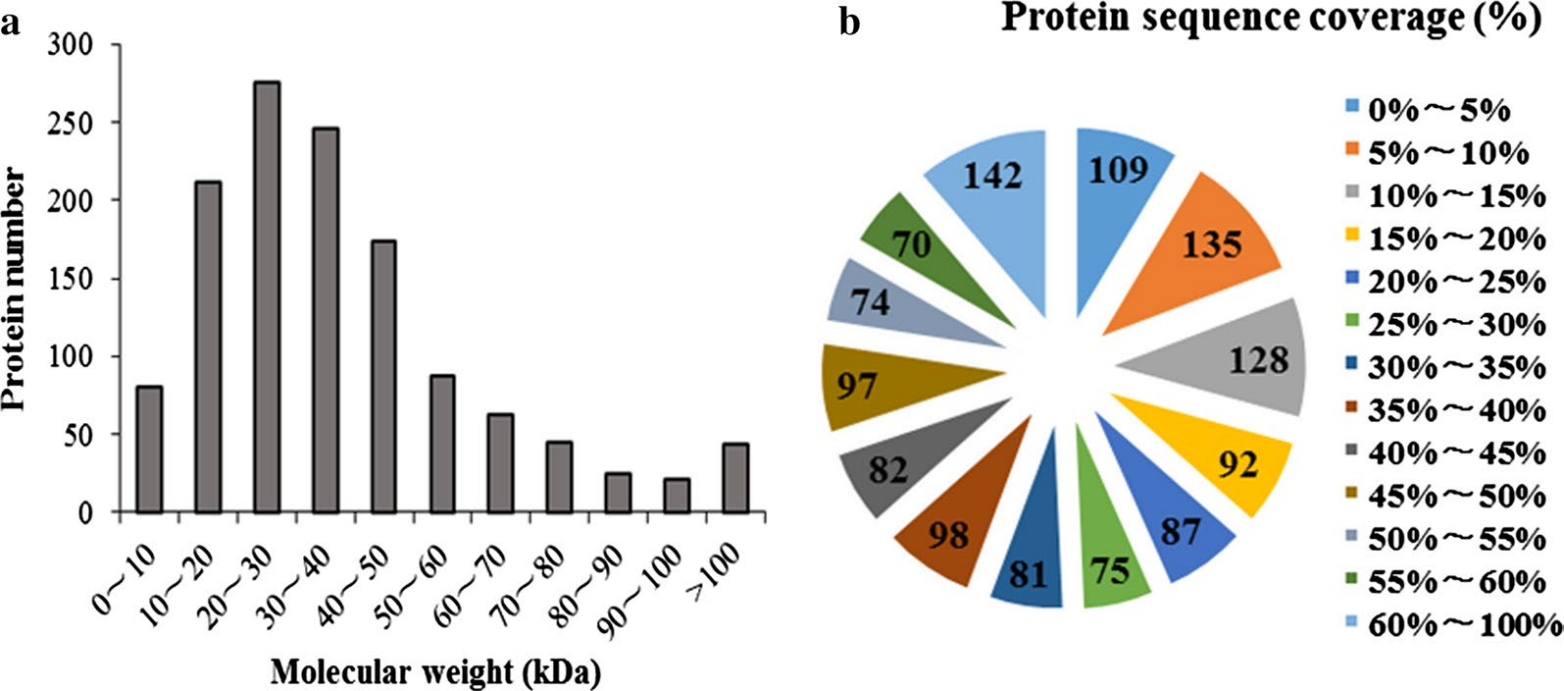
Overview of the proteomic analysis. **a** Molecular weight distribution of the identified proteins. **b** Sequence coverage of the identified proteins

In the present work, 1269 proteins were identified from analysis of three biological replicates. Figure 1a shows the molecular weight distribution of the identified proteins, Most had a molecular weight of 30–60 kDa, and these accounted for approximately 87% of all identified proteins. In addition, a few proteins with a molecular weight of greater than 100 kDa were identified, indicating that iTRAQ technology has a high detection sensitivity and can identify a wide range of proteins. Figure 1b shows the coverage of protein sequences. Most proteins had a peptide coverage of less than 60%, and 15–60% of proteins had a relatively uniform peptide coverage.

### Identification of differentially expressed proteins

**Fig. 2:**
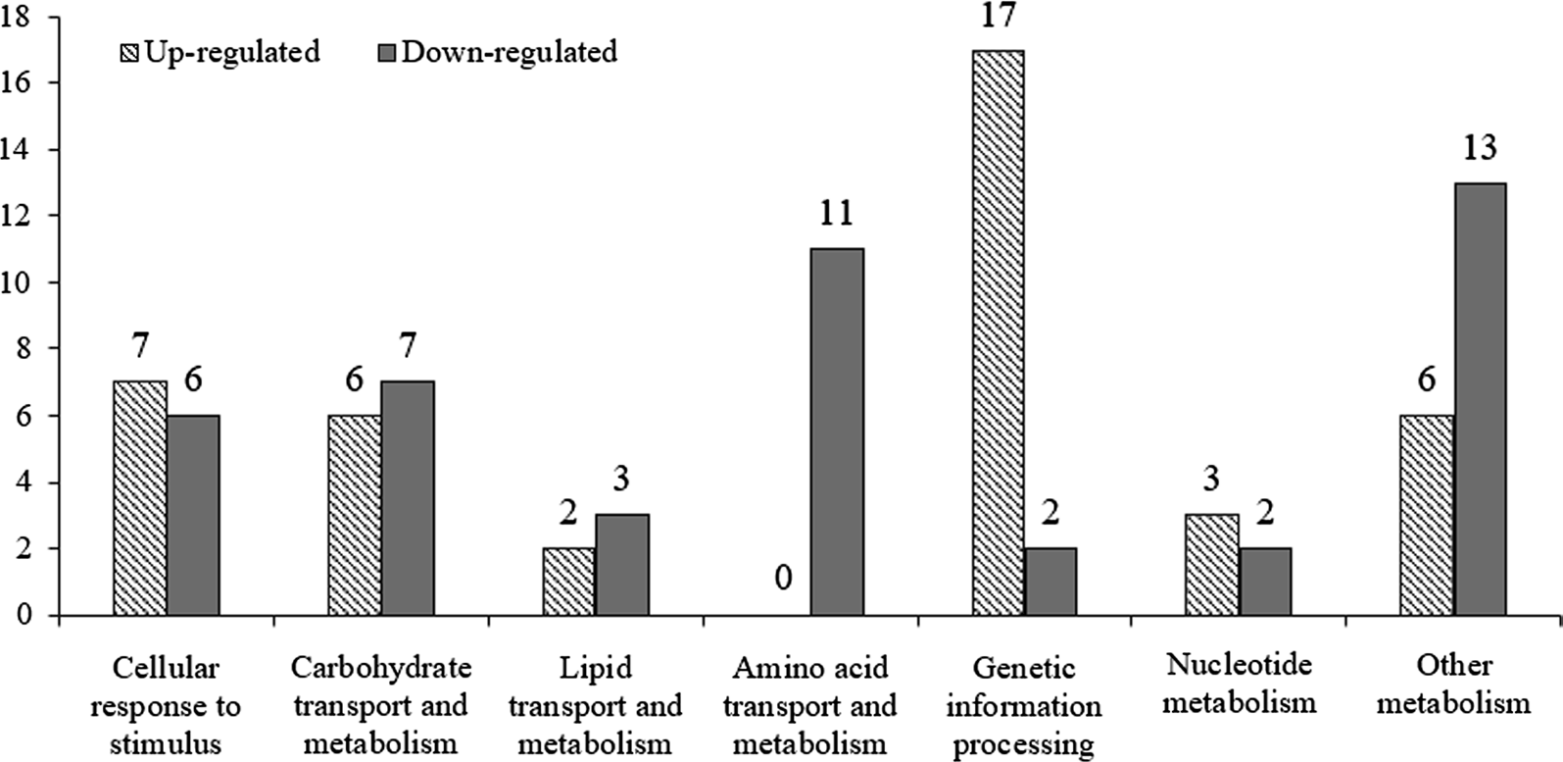
Functional categories of the differentially expressed proteins

In this work, 132 differentially expressed proteins were significantly affected by *ccpA* inactivation in the mutant strain compared to the parental strain, including 58 up-regulated proteins and 74 down-regulated proteins. Except for some unknown functional proteins, these differentially expressed proteins are listed in Table 1, and they were classified into the following categories: cellular response to stimulus, carbohydrate transport and metabolism, lipid transport and metabolism, amino acid transport and metabolism, genetic information processing, nucleotide metabolism, and other metabolism. As shown in Fig. 2, 13 differentially expressed proteins were associated with cellular response to stimulus, as well as carbohydrate transport and metabolism. 11 proteins were involved in amino acid transport and metabolism. 19 proteins were related to each of the terms genetic information processing and other metabolism. 10 proteins were involved in lipid transport and nucleotide metabolism. These data suggested that CcpA, as a pleiotropic regulator, was involved in multiple physiological and metabolic processes.

**Table 1:** List of partial differential expression proteins identified by the iTRAQ analysis

Accession	Protein description	MW [kDa]	Coverage	Fold change T/C^a^
Cellular response to stimulus				
F0HVQ0	SOS regulatory protein LexA	22.16	2.91	2.228
A0A061BVN8	Regulatory protein RecX	30.45	3.32	2.106
S2KUP9	Transcriptional regulator	34.81	1.92	1.872
A0A061BU68	Transcriptional regulator (Crp family)	35.63	3.43	1.714
A0A061CJZ0	Transcriptional regulator (MarR family)	13.13	17.39	1.636
A0A061CK68	Heat-inducible transcription repressor HrcA	39.09	4.03	1.602
A0A061BU38	DNA repair protein RecO	27.59	6.83	1.524
Q1G937	GroEL chaperonin	57.30	78.77	− 1.592
A0A061CKD6	Chaperone protein DnaK	66.02	64.50	− 1.600
A0A061C1K9	Response regulator	27.86	55.60	− 1.715
Q048Y2	GroES chaperonin	10.27	85.11	− 1.802
A0A061BLC4	Heat-shock protein Hsp20	15.79	56.03	− 2.288
G8I2M0	Translation elongation factor Tu	9.71	34.44	− 2.985
Carbohydrate transport and metabolism				
A0A061BX52	EpsIIH, glycosyltransferase	42.16	18.01	1.802
Q1G8F3	EpsIM, glycosyltransferase	40.55	24.17	1.744
Q1G8E9	EpsIH, glycosyltransferase	31.48	28.09	1.624
Q1G7Z6	Pyruvate oxidase	67.67	64.94	1.592
Q1G8E8	EpsIG, glycosyltransferase	19.19	38.92	1.529
Q1GAY6	Acetate kinase	43.16	46.08	1.512
Q71HT0	Phosphopyruvate hydratase	15.15	82.19	− 1.520
A0A061BLT3	Galactosyltransferase	39.99	9.02	− 1.567
A0A061CQG7	Phosphoglycerate kinase	42.71	80.89	− 1.678
D8FNY3	Pyruvate kinase	62.90	53.99	− 1.949
A0A061BZB8	6-Phosphofructokinase	38.76	54.87	− 2.268
D8FN74	d-lactate dehydrogenase	36.98	56.46	− 2.283
D8FM36	Ribose-phosphate diphosphokinase	36.91	42.22	− 3.636
Lipid transport and metabolism				
A0A061BKY2	Acyl-phosphatelycerol 3-phosphate acyltransferase	29.91	2.33	1.579
A0A061BRJ8	Glycerophosphoryl diester phosphodiesterase	26.73	16.96	1.500
Q1G9C4	Putative acylphosphatase	10.05	46.67	− 1.522
A0A061C1U5	Geranylgeranyl pyrophosphate synthase	32.95	35.14	− 1.667
G6EWT6	Cyclopropane-fatty-acyl-phospholipid synthase	44.22	44.16	− 2.247
Amino acid transport and metabolism				
A0A061BW11	Cysteine–tRNA ligase	53.65	37.97	− 1.534
D8FQC5	Arginine–tRNA ligase	64.10	58.05	− 1.610
A0A061BMI1	Homoserine *O*-succinyltransferase	29.79	13.85	− 1.618
Q1GBX1	Amino acid ABC transporter, substrate binding protein	31.98	55.59	− 1.626
Q04CP9	*S*-ribosylhomocysteine lyase 1	17.85	68.55	− 1.672
A0A061BL94	Amino acid ABC transporter, ATP-binding protein	28.25	52.44	− 1.818
A0A061BUC1	Cystathionine gamma-synthase	43.06	44.67	− 1.832
G6EWC8	Serine protease	43.42	3.53	− 1.873
Q1G9F7	Phosphoribosylformylglycinamidine synthase subunit PurQ	24.22	11.61	− 2.165
A0A061BUK8	Cysteine synthase	32.43	58.31	− 2.174
A0A0D6ZH63	Amino acid ABC transporter permease	25.34	12.95	− 2.252
Genetic information processing				
Q1GBK0	50S ribosomal protein L30	6.67	60.66	3.435
Q04BZ9	50S ribosomal protein L18	12.92	73.95	2.171
Q71J17	50S ribosomal protein L22	10.58	68.37	2.147
G6F488	Ribosome maturation factor RimP	15.52	11.28	1.949
D8FNZ8	RNA polymerase sigma factor	42.76	30.42	1.760
Q049M4	50S ribosomal protein L21	11.33	61.17	1.693
Q1GBK4	30S ribosomal protein S8	14.44	70.45	1.689
A0A061CI05	30S ribosomal protein S14	7.09	11.48	1.687
Q1G904	50S ribosomal protein L10	18.18	70.41	1.647
Q1GBL8	50S ribosomal protein L3	22.75	73.68	1.598
Q1GBI6	50S ribosomal protein L13	16.38	73.47	1.581
F0HX40	50S ribosomal protein L24	8.21	58.23	1.572
F0JZX6	30S ribosomal protein S17	10.49	45.45	1.542
G6EVN1	50S ribosomal protein L4	21.87	43.72	1.529
Q04BZ2	50S ribosomal protein L36	4.41	26.32	1.517
Q1G7Z4	tRNA modification GTPase MnmE	49.93	32.32	1.510
Q1G905	50S ribosomal protein L12	12.34	59.50	1.506
F0HW20	50S ribosomal protein L34	5.41	13.04	− 1.529
Q1G8Z5	50S ribosomal protein L33	5.49	20.41	− 2.141
Nucleotide metabolism				
A0A061BND	Cytidine deaminase	15.43	4.32	2.065
Q1GA92	Pseudouridine synthase	33.87	11.22	1.955
G6F8G6	*N*-acetylglucosaminyldiphosphodolichol *N*-acetylglucosaminyltransferase	15.54	12.12	1.676
F0JZF8	Inosine-5′-monophosphate dehydrogenase	40.13	79.74	− 1.534
A0A061C7C2	Putative pyridine nucleotide-disulphide oxidoreductase	48.70	59.69	− 1.560
Other metabolism				
A0A0D6ZHP8	Glutamine transporter, ATP-binding protein	27.16	10.16	1.714
F0HUZ5	FeS cluster assembly scaffold IscU	15.81	18.49	1.691
Q1G9D0	membrane protein	93.97	1.68	1.635
A0A061BP48	Thioredoxin	12.00	79.25	1.542
Q1GAT1	Cell division protein SepF	15.63	62.32	1.534
Q1GAK5	Segregation and condensation protein B	22.21	39.00	1.528
A0A061BY60	Hydrolase (NUDIX family)	19.83	8.62	− 1.513
A0A061BWU4	ABC transporter, ATP-binding protein	23.77	53.30	− 1.572
Q1G869	ATP-dependent Clp protease, ATP-binding subunit clpL	76.90	76.87	− 1.600
D8FM00	Phosphonate ABC transporter, substrate-binding protein	34.08	46.33	− 1.603
D8FPT9	ATP-dependent Clp protease, ATP-binding subunit clpE	76.96	61.78	− 1.637
G6EW12	Peptide hydrolase	51.31	31.28	− 1.650
A0A061BU80	Cation transporting P-type ATPase	81.71	29.80	− 1.653
A0A061BUM1	Phosphoribosylglycinamide formyltransferase	21.64	10.88	− 1.658
A0A061BVN4	2,5-didehydrogluconate reductase	31.01	26.09	− 1.678
A0A061CDM4	X-Pro dipeptidase PepQ	41.09	48.10	− 1.783
D8FLL0	ATP synthase subunit beta	52.10	60.33	− 1.832
Q71IA3	3-Hydroxy-3-methylglutaryl-coenzyme A reductase	15.95	24.16	− 1.852
A0A061CI27	ATP-dependent Clp protease, ATP-binding subunit clpC	90.83	48.60	− 2.169

^a^Average fold-change was calculated as the ratio of the CcpA-negative mutant of *L. bulgaricus* ATCC11842 to the parental strain for up-regulated proteins and as the negative reciprocal values for down-regulated proteins, Proteins with the fold-change of ≥ 1.5 or ≤ − 1.5 and P-value < 0.05 were considered to be significantly up-regulated or down-regulated, respectively, based on statistic analysis for three biological replicates [19]

**Fig. 3:**
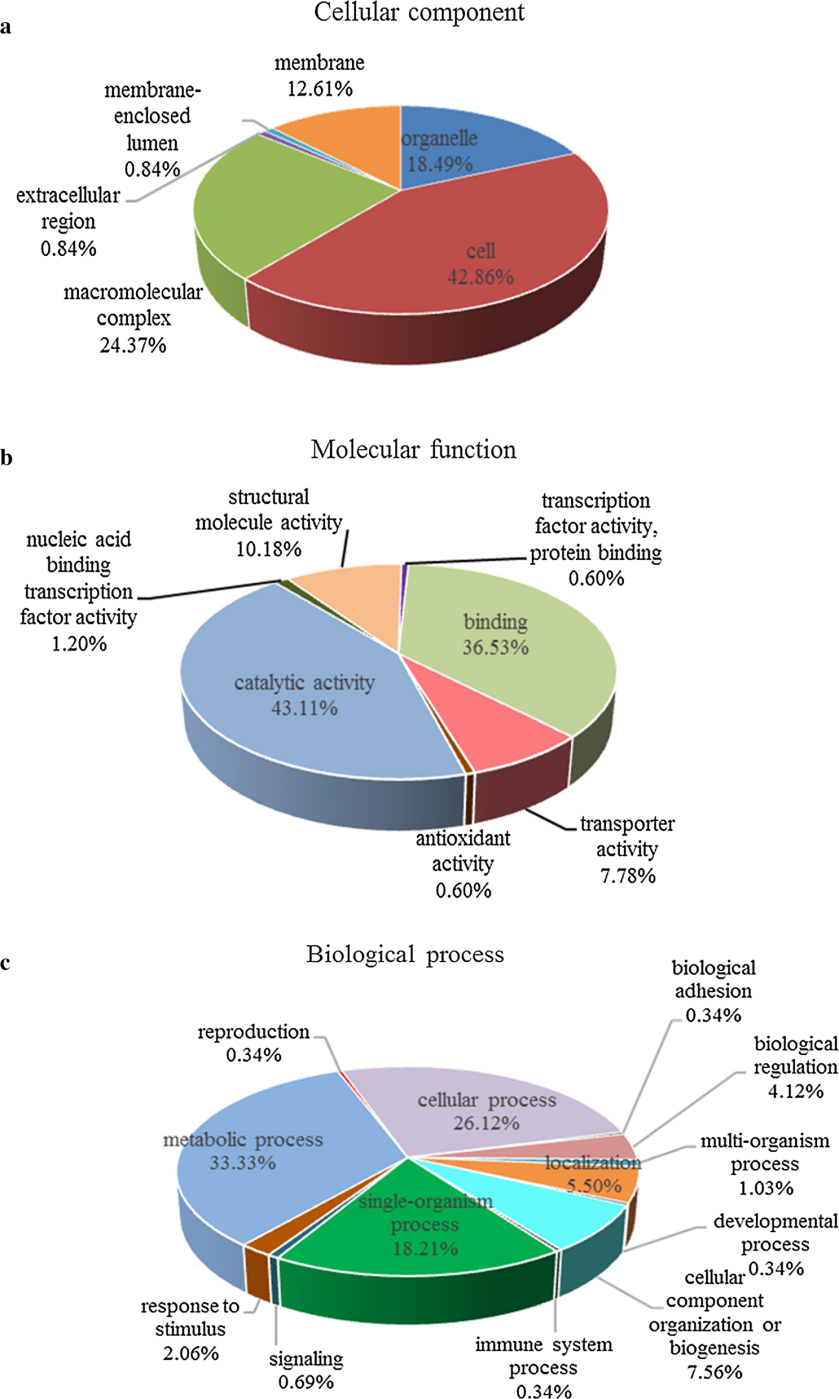
GO category assignment of the differentially expressed proteins

The results of the protein Gene Ontology (GO) analysis are shown in Fig. 3. Regarding cellular components, 43% were related to cell parts and 24% were related to macromolecular complexes; followed by organelles (18%) and cell membranes (13%) (Fig. 3a). Catalytic activity (43%) and binding (36%) were the dominant terms in the molecular function category. In addition, some proteins were related to transporter activity (8%) and structural molecule activity (10%) (Fig. 3b). In the biological process category, the differentially expressed proteins were related to metabolic process, cellular process, response to stimulus, immune system process, biological adhesion, and other such terms. The most enriched biological process terms were metabolic process and cellular process, which respectively accounted for 33.3% and 26.1% of the total biological process, followed by single-organism processes (18.2%) (Fig. 3c).

Kyoto Encyclopedia of Genes and Genomes (KEGG) pathway analysis of the differentially expressed proteins showed that 15 proteins were associated with the ribosome. 8 proteins were enriched in the biosynthesis of amino acids pathway, and 6 were annotated in the cysteine and methionine metabolism pathways, as well as the purine metabolism pathway. A total of 15 proteins were annotated in the carbon metabolism, glycolysis/gluconeogenesis and pyruvate metabolism pathways. Additionally, lower numbers of proteins were related to pathways such as ABC transporters, aminoacyl-tRNA biosynthesis, and phosphotransferase system. (Additional file 1: Table S1).

### Gene transcription analysis

**Fig. 4:**
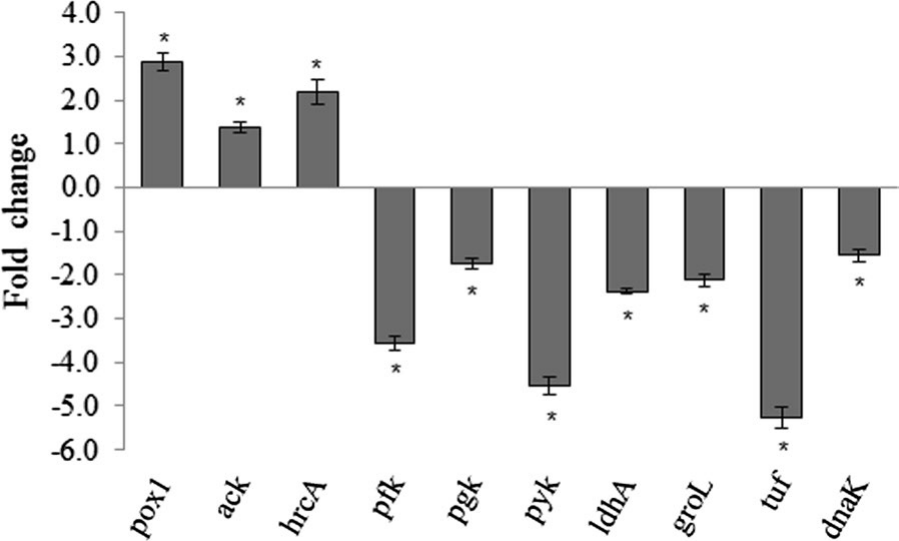
Relative expression levels of carbohydrate metabolism and stress response related genes as measured by qRT-PCR. Relative expression fold changes were calculated relative to the transcript levels in the *ccpA*-inactivated mutant strain compared to the parental strain for up-regulated genes and as the negative reciprocal values for down-regulated genes. The data were normalized to the transcription level of 16S rRNA and are expressed as the mean ± SD of three biological and technical replicates. Statistical significance is identified as *P < 0.05

To verify the proteome results, 10 key genes were evaluated by quantitative real-time PCR. The tested genes involved in carbohydrate metabolism, namely encoding phosphofructokinase (Pfk), pyruvate kinase (Pyk), D-lactic dehydrogenase (LdhA), phosphoglycerate kinase (Pgk), pyruvate oxidase (Pox) and acetate kinase (Ack), are shown in Fig. 4. The expression of *pox1* was significantly up-regulated 2.87-fold, and *ack* was up-regulated 1.37-fold in the mutant strain compared with the parental strain (P < 0.05). In contrast, the gene expression levels of *pfk*, *pgk, pyk* and *ldh*A decreased by 3.57-, 1.75-, 4.55- and 2.38-fold, while the iTRAQ results showed that the protein expression levels of Pfk, Pgk, Pyk and LdhA decreased by 2.27-, 1.68-, 1.95-, and 2.28-fold, respectively (P < 0.05). In addition, genes involved in the stress response, namely encoding elongation factor Tu (EF-Tu), molecular chaperone Dnak (DnaK), chaperonin GroEL (GroEL), and heat-inducible transcription repressor HrcA (HrcA), were evaluated (Fig. 4). The expression of *hrcA* was up-regulated 2.17-fold, while that of *Tuf, dnaK,* and *groL* were decreased by 5.26-, 1.56-, and 2.13-fold, respectively. These results indicated that the protein expression levels were consistent with the corresponding mRNA expression levels, suggesting these proteins were regulated mainly at the transcriptional level in cells.

## Discussion

Aerobic growth significantly affect the energy metabolism and stress tolerance of LAB with important consequence in food-related application [23]. Aerobic cultivation, moreover, support the activation of respiratory chain in LAB, resulting in phenotypes with improved growth and technological performance [23]. The global regulation of CcpA in aerobic metabolism and stress response has been studied in several important LAB in the dairy industry [11, 15]. However, related literature in *L. bulgaricus* is scarce. Therefore, the role of the global regulator CcpA in *L. bulgaricus* and its *ccpA* deletion mutant strain was investigated in the present study from the differential proteomic perspective. A physiological and metabolic study (growth, metabolite production and stress tolerance) previously performed on the same experimental system [22], appropriately integrated to provide deeper insight into the cellular processes mediatized by CcpA in *L. bulgaricus*.

In this work, inactivation of *ccpA* caused changes in the expression of some proteins related to stress response in cells. Among the differentially expressed proteins, heat shock protein (Hsp20), cold stress protein (Csp), elongation factor Tu (EF-Tu), and molecular chaperones (GroEL, GroES, and DnaK) were down-regulated in the mutant strain compared to the parental strain, while the transcriptional repressor protein HrcA, the DNA repair protein RecO, the regulatory protein RecX and the SOS regulatory protein LexA were up-regulated.

The expression of HrcA was up-regulated in the mutant strain, showing that it was negatively regulated by CcpA in *L. bulgaricus*. HrcA is a negative regulator of the class I heat shock gene (*dnaK* and *groELS* operons) that prevents these operons from inducing heat shock [24]. As expected, the expression of DnaK and GroEL was down-regulated in the mutant strain, indicating that these operons were activated by CcpA. Our previous studies have found that *ccpA* inactivation reduced the heat tolerance of *L. bulgaricus* [22], which was consistent with the proteomic data. The results were in agreement with studies on *L. plantarum* with *ccpA* inactivation [11].

EF-Tu not only promotes the binding of aminoacyl-tRNA to ribosomes but also participates in protein folding and protects cells against environmental stress. Upregulation of the elongation factor EF-G in *L. bulgaricus* improved its tolerance to salt stress [25]. In *L. plantarum*, the expression of EF-Tu was found to be reduced by inactivation of *ccpA* [11], consistent with our data.

Our previous study showed that aerobic growth significantly enhanced tolerance to heat and oxidative stress in *L. bulgaricus* and its *ccpA* deletion mutant strain [22]. This enhancement may have practical significance for the production of starter, for instance, although it is far more economic than freeze drying, spray drying imposes oxidative damage and stress on cells [26].

Thioredoxin (Trx) is an important protein that participates in many redox reactions and regulates the function of some enzymes in microorganisms. A transcriptomic study suggested that the *trxA2* and *trxB1* genes play a key role in the oxidative stress response mechanism of *L. plantarum* [27], the expression levels of genes related to the stress response and sulfur-containing amino acid biosynthesis were significantly affected by the overexpression of *trxB1*. Studies on the aerobic growth of *L. lactis* have shown that Trx also was involved in carbon and lipid metabolism [28]. Zotta et al. [10] reported that the expression of NADH oxidase and Pox was significantly up-regulated in *L. plantarum,* which was considered to be the main reason for the improved oxidative tolerance of the strain after *ccpA* knockout. In this study, the expression of Trx and iron–sulfur (Fe–S) were significantly increased in the mutant strain due to *ccpA* inactivation; thus, the expression of these proteins was negatively regulated by CcpA. As previous demonstrated, the mutant strain was more tolerant than the parental strain for oxidative stress [22]. We speculate that these up-regulated proteins involved in redox reactions may enhance the oxidative tolerance of the mutant strain. Collectively, these results indicated that CcpA acts as a pleiotropic regulator in coordinating oxygen, iron and carbon metabolism [14].

The role for CcpA in control of carbohydrate metabolism has been reported in some LAB [12, 29]. The regulatory pathways differ among microorganisms, although all are based on PTS/CcpA-mediated signal transduction [30]. In *L. lactis* and *L. plantarum*, the *pfk*-*pyk* operon is positively regulated by CcpA, which activates gene transcription [9, 14]. However, in *L. casei*, CcpA negatively regulates the expression of the *pfk* and *pyk* genes [12]. The *pfk*-*pyk* operon in *L. bulgaricus* is regulated by CcpA, but the regulatory mechanism is unclear. Our previous work showed that the activities of key enzymes (Ldh, Pyk, and Pfk) in the glycolytic pathway are significantly decreased by *ccpA* inactivation [22]. In this study, Pfk, Pyk and LdhA were found to be down-regulated at the protein expression level in the mutant strain compared with the parental strain; additionally, changes in their gene transcription levels were consistent with those in the protein expression levels. Thus, our results demonstrated that CcpA positively regulated the *pfk*-*pyk* operon in *L. bulgaricus*.

**Fig. 5:**
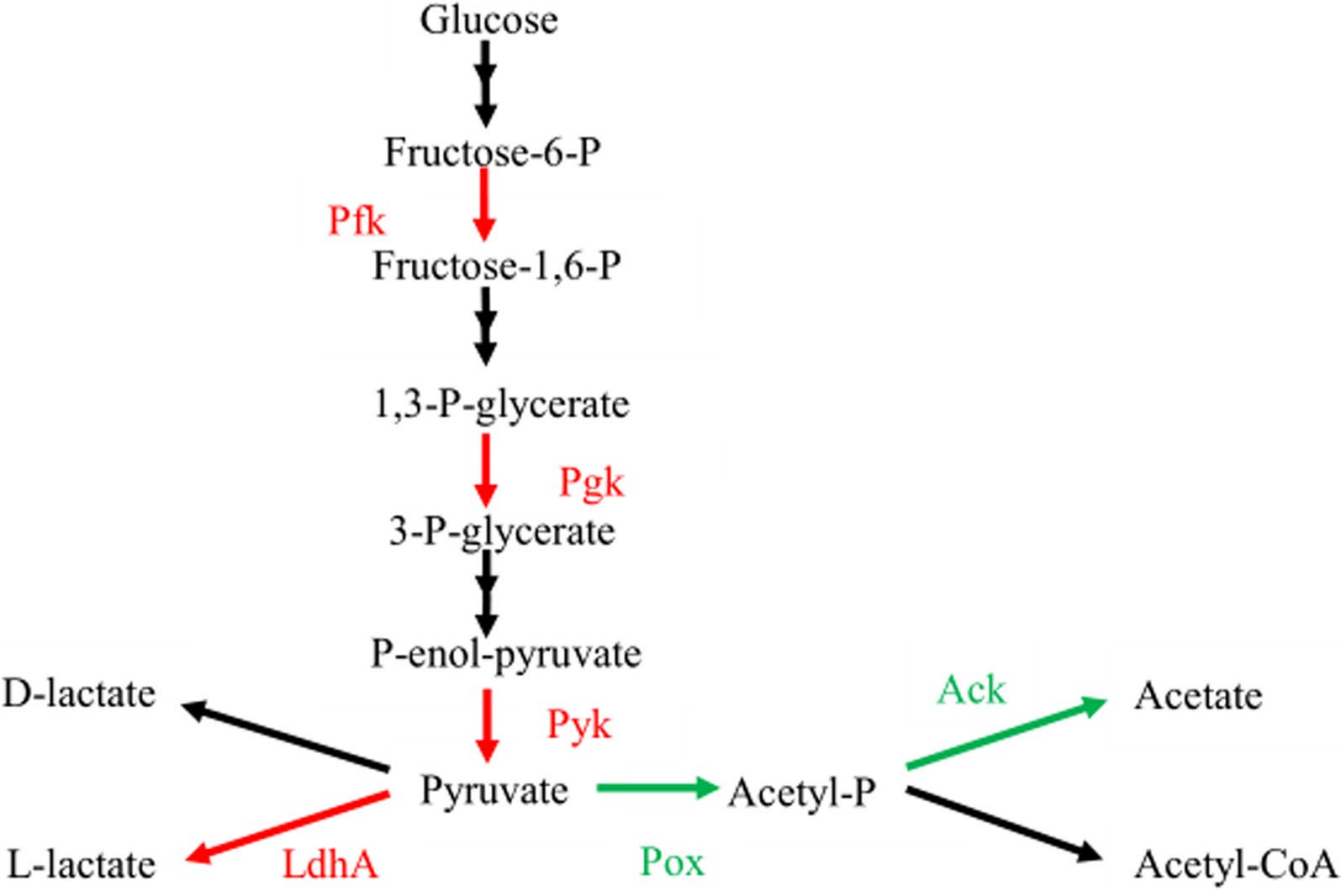
Overview of the metabolic pathway of lactate and acetate production in *L. bulgaricus.* The red arrows indicated proteins were down-regulated by *ccpA* inactivation in *L. bulgaricus*, as observed at the protein level and transcriptomic level. The green arrows indicated proteins that were up-regulated. *Pfk* 6-phosphofructokinase, *Pgk* phosphoglycerate kinase, *Pyk* pyruvate kinase, *LdhA* d-lactate dehydrogenase, *Pox* pyruvate oxidase, *Ack* acetate kinase

CcpA directly or indirectly controls the expression levels of enzymes involved in the pyruvate pathway, which facilitate the utilization of available carbohydrates [9, 30] Pyruvate plays a very important role in growth and metabolism since it affects the energy and redox status of the cells [30]. In this work, inactivation of *ccpA* caused pyruvate flux away from lactate production in *L. bulgaricus* under aerobic conditions. As shown in Fig. 5, the expression of some enzymes involved in glycolysis was significantly down-regulated while that of Pox and Ack was significantly up-regulated. Previous work on growth and metabolite production also showed decreases in the growth rate, the utilization of glucose, the production of lactic acid, and increases in the yield of acetic acid in the mutant strain [22], which verified our proteomic results. Collectively, these findings clearly showed that CcpA mediated the shift from homolactic fermentation to mixed acid fermentation in *L. bulgaricus*, which was in agreement with the findings of Zotta et al. [10] and Mazzeo et al. [11] for *L. plantarum.*

Inactivation of *ccpA* altered the expression of some proteins involved in protein synthesis and translation in *L. bulgaricus*. For example, the expression levels of various ribosomal genes were up-regulated in the mutant strain. Many studies have showed that upregulation of ribosomal in LAB under stress conditions, indicating that these ribosomes not only are involved in protein synthesis but also may be sensors for environmental changes [31]. Clp proteases belong to the family of ATPases, which plays a key role in refolding or degrading damaged proteins in low-GC Gram-positive bacteria [32]. In this study, the expression of multiple Clp proteases was down-regulated in the mutant strain compared to the parental strain, similar to the study of the *ccpA* mutant strain of *L. plantarum* [10].

Remarkable changes in the proteome of *L. bulgaricus* were caused by the *ccpA* inactivation, thus confirming its role as a pleiotropic regulator under acerbic conditions. Moreover, the role of CcpA in aerobic growth was also reported by Mazzeo et al. [11] in *L. plantarum* compared with anaerobic growth at proteomic level, which brought new insights into the regulatory mechanism of CcpA response to oxidative stress. Therefore, comparison with anaerobic growth could provide a novel direction for our future research.

## Conclusions

This work is the first report of the role of CcpA in regulating carbohydrate metabolism and stress response of *L. bulgaricus* under acerbic conditions at proteomic level. The proteomic results coherently match with physiological and metabolic features previous demonstrated by Li et al. [22] on the same experimental system. A total of 132 proteins were identified to be differentially expressed between the mutant strain and the parental strain. Inactivation of *ccpA* negatively affected the expression of key enzymes involved in glycolysis, supporting the conclusion that CcpA mediated the shift from homolactic fermentation to mixed acid fermentation in *L. bulgaricus*. This proteomic results provide new knowledge in the role of global regulator CcpA in *L. bulgaricus*, and may have practical significance for the production of starter and probiotic cultures.

## Materials and methods

### Bacterial strains and growth conditions

*Lactobacillus bulgaricus* ATCC11842 strain was purchased from the American Type Culture Collection (Manassas, VA, USA). Inactivation of the *ccpA* gene was achieved by homologous recombination as described previously by Li et al. [23]. Both strains were stored at − 80 °C in 20% glycerol in this experiment. *L. bulgaricus* and its mutant *ccpA* deletion strain were cultivated at 37 °C in MRS broth under aerobic conditions in Erlenmeyer flasks (the medium volume was 1/10 of the flask volume) with shaking (150 rpm). Bacterial samples were harvested during exponential growth phase (final OD_600_ = 1.0) and immediately frozen in liquid nitrogen until further use [23].

### Protein preparation

Total protein extraction was performed as previously reported with slight modifications [33]. Briefly, all strains were cultivated overnight in MRS broth at 37 °C and harvested by centrifugation at 8000×*g* for 20 min at 4 °C. After the cell pellets were washed with 50 mM Tris buffer, 1 mL of lysis buffer (7 M urea, 2 M thiourea, 4% CHAPS, 1 mM DTT, 1 mM PMSF, and 2 mM EDTA) was added, and the samples were sonicated in an ice bath until the bacterial solution was clarified. Then, the samples were transferred into tubes and centrifuged at 20,000×*g* for 20 min at 4 °C. Then, the supernatant was collected for SDS-PAGE on 5% stacking gels and 12.5% resolving gels. Gels were stained with Coomassie Blue. Protein concentrations were quantified using the Bradford method.

### Protein digestion and iTRAQ labeling

Protein digestion was performed using the FASP method [34]. Briefly, 100 μg of each sample was added to precooled acetone (volume ratio of acetone:sample = 6:1), and the mixture was precipitated for 1 h at − 20 °C. The protein precipitate was fully dissolved using dissolution buffer (50 μL) and 1% SDS (1 μL) from the iTRAQ kit (Applied Biosystems, Foster City, CA, USA). Next, 4 μL of reducing reagent was added and reacted at 60 °C for 1 h. Then, 2 μL of cysteine-blocking reagent was added at room temperature for 10 min in the dark. The samples were transferred to new ultrafiltration tubes and centrifuged at 12,000×*g* for 20 min. After the lower layer of solution in the collection tubes was discarded, 100 μL of dissolution buffer was added and centrifuged under the same conditions described above. This step was repeated three times. The collection tubes were replaced with new tubes, and 4 μg of trypsin was added to the ultrafiltration tubes and incubated overnight at 37 °C. The enzymatically decomposed peptides were collected by centrifugation, and 50 μL of dissolution buffer was added to the ultrafiltration tubes. The lower layer of solution was then collected and combined with the previously obtained solution. The peptides from each sample were desalted, and the protein concentration was determined by measuring the OD at 280 nm. iTRAQ reagent was dissolved in 60 μL of isopropanol according to the manufacturer’s instructions and then mixed with the samples at room temperature for 2 h. All samples were combined after labeling and dried by vacuum centrifugation for strong cation exchange (SCX) fractionation.

### SCX fractionation

iTRAQ-labeled peptides were fractionated by SCX chromatography according to the experimental conditions described previously [34]. Configured transfer buffer A (10 mM KH_2_PO_4_ and 25% acetonitrile, pH 3.0) and elution butter B (500 mM KCl, 10 mM KH_2_PO_4_, and 25% acetonitrile, pH 3.0) were used for liquid chromatography. The samples were dissolved in 4 mL of buffer A and loaded into a 4.6 × 100 mm polysulfoethyl column containing 5-µm particles (PolyLC Inc., Columbia, MD, USA) in a sampler for separation. The column was equilibrated in buffer A for 10 min. Peptides were eluted with buffer B at a flow rate of 1 mL/min as the following gradient: 0–8% buffer B for 20 min, 8–52% buffer B for 25 min, 52–100% buffer B for 3 min, 100% buffer B for 7 min and then buffer B was reset to 0% for 5 min, for a total of 60 min. Based on the UV absorbance at 214 nm, the fractions were collected in 1 min intervals using a collector and then vacuum dried for subsequent analysis.

### Liquid chromatography/tandem mass spectrometry (LC–MS/MS) analysis based on Q Exactive technology

Mass spectrometry analysis was carried out as described by Luo et al. [35] with moderate modifications. Each fraction was dissolved in buffer C (0.1% formic acid) and then analyzed using the EASY-nLC 1000 HPLC system (Thermo Fisher Scientific, Waltham, MA, USA). A total of 10 μL of the peptide mixture was injected onto a PepMap C18 trap column (100 μm × 2 cm, 3 μm, 100 Å, Thermo Fisher Scientific, Rockford, IL, USA) via a sampler and were then separated on an Acclaim PepMap C18 column (75 μm × 10 cm, 3 μm, 100 Å, Thermo Fisher Scientific) at a flow rate of 300 nL/min with an elution gradient of 0–50% buffer D (84% acetonitrile and 0.1% formic acid) for 55 min and 50–100% buffer D for 55–57 min. Buffer D was maintained at 100% after 57 min.

MS data were acquired using a data-dependent top10 method in the mass spectrometer, which was operated in positive ion mode with a mass range of 300–1800 m/z for high-energy collisional dissociation (HCD) fragmentation. The MS spectra and HCD spectral scans were acquired at a resolution of 70,000 and 17,500 at m/z 200, and the maximum injection times were set to 10 and 60 ms, respectively. The instrument was operated using a peptide recognition pattern. The other identification parameters were as follows: a normalized collision energy of 30 eV, an isolation window of 2 m/z, a dynamic exclusion duration of 40 s, and an underfill ratio of 0.1%.

### Protein identification and data analysis

The raw mass spectrometry data were submitted to Mascot 2.2 software (Matrix Science, London, UK) through Proteome Discoverer 1.4, and searches were performed against the *L. bulgaricus* database, which contains 24,177 protein sequences, for protein identification and quantification. The screening conditions for the trusted proteins included a false discovery rate of no more than 1%, which was calculated with a decoy database model, and the protein ratios were quantified based on the median of only unique peptide ratios in Mascot. The t-test was used to evaluate statistical significance. Proteins with the fold-change of ≥ 1.5 or ≤ −1.5 and P-value < 0.05 were considered significantly up-regulated or down-regulated, respectively. The Blast2GO program was used to annotate the molecular function, biological process and cellular component information for the target proteins. Metabolic pathway analysis of the differentially expressed proteins was performed using KEGG.

### RNA extraction and transcriptional analysis (qRT-PCR)

Bacterial cells were harvested by centrifugation at 8000×*g* for 20 min at 4 °C as described above. RNA extraction and complementary DNA (cDNA) synthesis were separately performed using the RNeasy Midi Kit (Tiangen, Beijing, China) and cDNA RT reagent kit (Taraka, Dalian, China) according to the manufacturer’s protocol. The sequences of the primers are listed in Additional file 1: Table S2. qRT-PCR was performed with Taq SYBRGreen qPCR Premix in a LightCycler instrument (ABI PRISM 7500 System, Applied Biosystems, Foster City, CA, USA) according to the manufacturer’s instructions. The thermal profile suggested by the manufacturer was 30 s of denaturation at 95 °C followed by 40 cycles of 10 s at 94 °C and 30 s at 60 °C. Three independent replicates of each sample were tested and the 2^−ΔΔCt^ method was used to calculate the expression levels of the target genes. For gene expression normalization, 16S ribosomal RNA (16S rRNA) was used as the internal standard for mRNA expression [36].

## Supplementary information

**Additional file 1: Table S1.** KEGG pathway analysis of differentially expressed proteins. **Table S2.** Primers used for qRT-PCR.

## Data Availability

All data generated or analyzed in this study are included in this published article and its additional file.

## Abbreviations

CcpA: catabolite control protein A
iTRAQ: isobaric tags for relative and absolute quantification
CCR: carbon catabolite repression
LAB: lactic acid bacteria
GO: gene ontology
KEGG: kyoto encyclopedia of genes and genomes
qRT-PCR: quantitative real-time polymerase chain reaction
Pfk: phosphofructokinase
Pyk: pyruvate kinase
Pgk: phosphoglycerate kinase
Pox: pyruvate oxidase
Ack: acetate kinase
EF-Tu: elongation factor Tu
EF-G: the elongation factor G
Hsp: heat shock protein
Csp: cold stress protein
Trx: thioredoxin
Fe–S: iron–sulfur
PTS: phosphotransferase system
OD: optical density
MRS: De Man–Rogosa–Sharpe
DTT: dithiothreitol
PMSF: phenylmethanesulfonyl fluoride
EDTA: ethylene diamine tetraacetic acid
SDS-PAGE: sodium dodecyl sulfate polyacrylamide gel electrophoresis
FASP: filter-aided sample preparation
SCX: strong cation exchange
LC-MS: liquid chromatography-mass spectrometry
HCD: high-energy collisional dissociation
