# Supplementary material for: Effects of ccpA gene deficiency in Lactobacillus delbrueckii subsp. bulgaricus under aerobic conditions as assessed by proteomic analysis
Source: Microb Cell Fact. 2020 Jan 13;19:9. doi: 10.1186/s12934-020-1278-7 (PMC6956489; doi:10.1186/s12934-020-1278-7)
Supplement: Supplementary file 1 — Additional file 1: Table S1. KEGG pathway analysis of differentially expressed proteins. Table S2. Primers used for qRT-PCR. [file 12934_2020_1278_MOESM1_ESM.docx]

**Table S1.**. KEGG pathway analysis of differentially expressed proteins.

| Pathway ID | Pathway Name | Number of proteins |
| --- | --- | --- |
| ko03010 | Ribosome | 15 |
| ko01200 | Carbon metabolism | 8 |
| ko01230 | Biosynthesis of amino acids | 8 |
| ko00270 | Cysteine and methionine metabolism | 6 |
| ko00230 | Purine metabolism | 6 |
| ko00010 | Glycolysis / Gluconeogenesis | 4 |
| ko00030 | Pentose phosphate pathway | 3 |
| ko00620 | Pyruvate metabolism | 3 |
| ko00920 | Sulfur metabolism | 3 |
| ko02010 | ABC transporters | 3 |
| ko00970 | Aminoacyl-tRNA biosynthesis | 2 |
| ko02060 | Phosphotransferase system (PTS) | 1 |
| ko00240 | Pyrimidine metabolism | 1 |
| ko00190 | Oxidative phosphorylation | 1 |
| ko00564 | Glycerophospholipid metabolism | 1 |

**Table S2.** Primers used in qRT-PCR.

| Name | Sequence (5′–3′) |
| --- | --- |
| 16S rRNA  *pgk*  *pfk*  *pyk*  *ldhA*  *ack*  *poxl*  *tuf*  *groL*  *dnaK*  *hrcA* | (F) ATCGGAAACTGTCATTCTTG  (R) CTAATCCTGTTCGCTACCC  (F) GCTGACTTGCCAGACGCTACTAC  (R) GAGATGCACCACCACCAGTTGAG  (F) AGCCGTAAGAGCCGTAACCAGAG  (R) TCAGGATAACGCGCAGAGTAGAGG  (F) CTAAGGTTCACGAAGGCGACATCC  (R) CAACACCGACAACAACTGGAATGC  (F) TGGCAGACAACGGCATCACTAAG  (R) AAGCCAAGTTCCTTAGCCTTAGCC  (F) ATGGCGTTATTGCAGACCTCAAGG  (R) GGTTGTGCAGAGGAGCGTAGTTAG  (F) AGAGAAGCAAGGCAAGCGTCAC  (R) CTTCGTCCTTCAAGTGGTCGTCAG  (F) GCCACGCCGACTACATCAAGAAC  (R) TTAACACCAACCTGACGAGCCAAC  (F) ACTGAACTGAGCGTGGTTGAAGG  (R) TCCATCTTGTCGTTGTCCGTTACC  (F) GCGGTTATCACTGTTCCAGCCTAC  (R) GTCGTCTTCATCCTTGTCCAAGCC  (F) TTGACTGACTACACGGCCATTGC  (R) TGACGCTGCCATCGCTGATTAC |
